# The perceptions of African women regarding natural menopause in Mamelodi, Tshwane district

**DOI:** 10.4102/curationis.v38i2.1531

**Published:** 2015-12-17

**Authors:** Gloria N. Makuwa, Steppies R. Rikhotso, Fhumulani M. Mulaudzi

**Affiliations:** 1 Department of Nursing Science, University of Pretoria, South Africa

## Abstract

**Background:**

The majority of South African aging population are women, who spend late adulthood experiencing natural menopause. Despite the government spending billions of rand on different services for ageing women, menopausal challenges to African women still receive little attention.

**Objectives:**

The aim of the study was to explore and describe the perceptions of African women regarding natural menopause, in order to propose recommendations for health and social support systems for women in Mamelodi, Tshwane district.

**Method:**

A qualitative, exploratory, descriptive and contextual design was used to conduct the study. The population of the study consisted of menopausal women, between the ages 45 and 60 years or more, visiting the clinics for collection of chronic medication and other health assessment. Individual face-to-face interviews were conducted, using a semi-structured interview guide to collect data. Tesch’s method of qualitative data analysis was used in the study.

**Results:**

The main theme that emerged from the study was ‘attitude toward menopause’, which was supported by cultural beliefs and experience. The African menopausal women expressed the importance of health support systems that will meet their needs within their context.

**Conclusion:**

Women’s health programs and educational health information at facilities should include menopausal education to promote and improve health of all African menopausal women during their adulthood. There is a need to establish a women’s health support group network within communities to share menopausal experiences with peers. The training and education curriculum of healthcare providers should include detailed menopause in order to provide comprehensive, congruent care.

## Introduction

Menopause is a normal, natural phenomenon for all ageing women, irrespective of culture or country of origin (Ramakuela et al. 2014:1). Rabiee, Nasirie and Zafarqandie (2014:4) explained menopause as a complex and multidimensional biological, social and psychological phenomenon for women between, on average, the ages of 45 and 60 (or more) years.

To promote women’s health during middle age and beyond, it is necessary to understand that menopause is a natural ageing process that all women undergo because of reduced production of reproductive hormones, oestrogen and progesterone (Lobo 2012; Rahman, Salehin & Iqbal 2011:188). There are a multitude of factors to be considered for menopause besides the women’s age; these include biological, socioeconomic and lifestyle factors. Rabiee et al. (2014:1) argued about the factors which affect the transitional period in a woman’s life and its relation to sexual desire in some women.

According to Norwitz and Schorge (2010:63), women experience menopause in a universal manner, as all bodily signs and symptoms are influenced by factors such as an ageing endocrine system leading to decline in ovarian function, a decrease in oestrogen that causes hot flushes, night sweats and vaginal dryness. However, the actual experience and attitude of women on menopause is individualistic. The attitude of women is ominously influenced by sociocultural factors – race, culture and ethnicity – as well as multicultural factors (Rapaport 2015:1). Agwu, Umeora and Ejikeme (2008:217) argued differently that not all women experienced menopause the same way, as their perceptions depend on their individual and sociocultural backgrounds.

In multicultural South Africa, health beliefs and practices vary within different communities. Menopause is viewed by some African women as the freedom from monthly menstruation and the transition period toward permanent infertility (Helman 2007:47; Ramakuela et al. 2014:2). Helman (2007:48) added that in the Zulu culture, for example, women who are in menopause are viewed as clean and can enter into the field or cows’ kraal without ruining the crops or causing sickness to the cattle. Ande et al. (2011:300) and Ozuzu-Nwaiwu (2007:34) support the view that most African women’s cultures accept menopause as a natural ageing process caused by a supernatural effects, resulting in African women rarely refusing hormone replacement therapy (HRT) (Ande et al. 2011:300; Ozuzu-Nwaiwu 2007:34), because of inadequate reproductive health-related knowledge about the phenomenon.

Some African women’s cultures perceive menopause as being a negative life event and a ‘disease-entrapment process’ resulting from cessation of menstruation, causing illness or poison in the woman’s body system. Limited literature has been conducted in the South African context to understand the perceptions of African women regarding menopause (Ozuzu-Nwaiwu 2007:35). As a result, the study was conducted to explore and describe the perceptions of African women regarding natural menopause in Mamelodi, Tshwane district.

### Problem statement

Women in multicultural South Africa perceive natural menopause differently as it is influenced by geographical location, cultural beliefs, socialisation, female reproductive health knowledge deficit and societal practices. Ramakuela et al. (2014:1) alluded that menopause in a rural village of Limpopo Province is determined by cultural beliefs because menopause is considered to be a taboo and/or sensitive discussion topic, meaning that women remain silent for fear of embarrassment (Agwu et al. 2008:220). Mustafa and Sabir (2012:176) revealed that other cultures, for example, Western women, report menopausal symptoms and receive assistance and support as they have reproductive health-related knowledge, including regarding ageing process. The cultural silence held by some African menopausal women may result in health-related problems because of inadequate knowledge. Approximately 51% of the South African population are women, with an average age of 55.2 years (Statistics South Africa 2012:3) and it is of great concern that few studies have been conducted that focus on understanding the cultural perceptions of South African women regarding reproductive health knowledge on natural menopause.

Currently, African women who are experiencing menopause reported health problems related to menopause only if they had a serious illness accompanied by unexplained pains, irritability, unusual emotional behaviour or unexplained signs and symptoms. The failure of some menopausal women to openly discuss and share information and reproductive health-related knowledge regarding natural menopause may lead to misunderstandings about menopause. There have been only a few studies done which are related to the perceptions of women regarding natural menopause.

#### Aim and objective of the study

The aim of the study was to explore and describe the perceptions of women regarding natural menopause in order to propose recommendations for healthcare providers to provide reproductive health-related knowledge regarding human developmental stages for all women in Tshwane district.

### Definition of concepts

#### Perceptions

The receiving, selecting, organising and interpreting of sensory information or stimuli by combining them with the results of previous experience to produce a meaningful picture of one’s world (Lantos 2010:388). It refers to how natural menopausal African women describe the experiences and feelings of being in the transitional period of their life, between the ages 45 to 60 years.

#### African woman

An ‘African woman’ is an adult woman who bears children and has a specified occupation or role in a family context such as a wife and/or mother or grandmother (Stevenson & Waite 2011:1659). In this study, it refers to an African woman between the ages of 46 and 55, experiencing natural menopause and residing in Mamelodi, Tshwane district. The African woman’s lifestyle and way of life is to focus on child bearing and caring for their large family (Hall et al. 2007:107).

#### Menopause

This refers to the condition designed by nature and regarded as a normal ageing process (Ramakuela et al. 2014:1), whereby the woman’s monthly menstrual periods ceases. It is normally diagnosed retrospectively a year following secondary amenorrhoea in woman of 45 years of age or older.

#### Significance of study

The findings of this study may assist in providing African women with health-related knowledge regarding menopause and contribute to the body of knowledge in nursing and other health professionals to provide comprehensive and congruent care to menopausal women of different African cultures. This could be achieved by sharing information through training institutions for the inclusion of the curriculum on women’s health, focusing on menopause. The findings will further inform the authorities to provide assistance to ageing African women for the establishment of health support groups in the community of Mamelodi in Tshwane district, where menopausal women share their menopausal experiences with peers.

## Research design

The research design is the overall plan for conducting research that will be applied in an attempt to address the research questions, including specifications for enhancing the study’s integrity (De Vos et al. 2011:309). A qualitative, explorative, descriptive and contextual design was used.

### Research approach and method

The design enhanced a systematic and interactive approach exploring life experiences of human participants to attach meaning (Polit & Beck 2012:16). The explorative aspect of the study was to explore the dimensions and relationships between the phenomena that were studied (Polit & Beck 2012:727); and the existing perceptions of African women regarding natural menopause were discovered through interviews and observations.

Descriptive study, according to Botma et al. (2010:194) is a study that is based on the general premises of naturalistic inquiry. The perceptions of African women regarding natural menopause were described within the context of the study setting. Contextual research is based on findings valid within the time, space and value context in which the study was being conducted (Polit & Beck 2012:732), in this case, in Mamelodi, Tshwane district.

Data were collected from African women who were at the menopausal stage of between the ages of 45 and 60 years, using semi-structured, individual interviews in Mamelodi, Tshwane district.

#### Context of the study

This article is written based on a mini-dissertation submitted for a Master’s degree at the University of Pretoria. The study was conducted in two Mamelodi clinics in Tshwane district.

#### Population, sample and sampling technique

African menopausal women were recruited during day clinic visits for minor ailments, routine body check-ups and for chronic medication collection, through the assistance of the clinic professional nurses at the setting. The purposive-convenience sampling technique was used to sample the participants (Botma et al. 2010:125). It is purposive as the researcher selected a representative sample of African menopausal women, namely, those between the ages of 45 and 60 years old.

The participants were purposively handpicked from the population (patients at the clinic) based on the researcher’s knowledge and judgement. It is also convenient, as the African menopausal women selected were only those who were at the clinic on the day of data collection, as assisted by the clinic professional nurses. The sample was drawn from the population according to the sample inclusion criteria, as described below.

#### Inclusion criteria

The inclusion criteria refer to those criteria that specify the population characteristics to be included in the study, that is, the eligibility criteria (Polit & Beck 2012:274). The sampling criteria for this study were:

- An ageing woman between 45 and 60 years old.
- Must have had no menstrual periods for the past 12 months.
- Must be attending the clinic services in Mamelodi, Tshwane district.
- Must reside in Mamelodi, Tshwane district.
- Must be willing to share their perceptions regarding menopause with the researcher voluntarily and without any coercion.

#### Data collection

The semi-structured, one-to-one individual interview method was used in the study to produce rich data as the participants were able to reflect and describe their perceptions regarding natural menopause in their own words.

#### Accesses to participants

The majority of the women were at the clinic to collect their chronic medication and, coincidentally, they met the inclusion criteria for the study. The main questions were asked by the researcher as guided by the interview guide and some probing questions followed. A digital recorder was used to record the interviews; and field notes were taken for non-verbal cues through observation by the researcher. Data were collected until data saturation was reached with the 18th participant, that is, when the menopausal women were providing information similar to the information already gathered from previous participants.

#### Data treatment

Data analysis is a process of bringing order and meaning to a large amount of data into smaller, manageable segments that can be retrieved and revised (De Vos et al. 2011:397). Data were analysed according to Tesch’ s (1990) eight steps of data analysis, as follows:

1. All data collected through interviews, digital recorder and observations were transformed into written text with the date, time and the place where the interview took place. Field notes were also typed and labelled.
2. The researcher read through the data several times to obtain a general sense of the information and to reflect on its overall meaning.
3. Several transcripts were read through and highlighted or segments (phrases) in them.
4. A list was compiled of all the topics that came to the researcher’s mind.
5. The researcher used the compiled list to analyse the transcripts by looking for segments (phrases) from the transcripts that fit the topics.
6. All the segments that fitted a particular topic were put together and were given descriptive names as subthemes.
7. The subthemes were sorted and grouped together, then given descriptive names as themes.
8. Digital recorder interviews were transcribed verbatim, then analysed and clustered into themes, categories and subcategories.

## Ethical considerations

The study obtained ethical clearance from the University of Pretoria’s Ethics Committee (protocol number 32/2014), the Ethical Committee of the Gauteng Department of Health, Tshwane district and the Nursing management of the two clinics in Mamelodi, Tshwane district. The recruited participants were provided with information leaflets and the researcher explained the aims, objectives and significance of the study. They were reassured that no harm would be experienced, either emotionally, physically or psychologically. The participants were given informed consent forms to sign voluntarily and without coercion. Confidentiality and anonymity were maintained throughout the report (De Vos et al. 2011:116; Polit & Beck 2012:177) and transcripts and recordings were kept in a locked safe by the researcher.

## Trustworthiness

The following criteria were used and applied throughout the study to ensure trustworthiness: credibility, dependability, transferability and confirmability (Botma et al. 2010:232):

- **Credibility** was enhanced by using member checking and peer examination. The researcher conducted semi-structured, one-to-one individual interviews until data saturation was reached, using digital recorder and field notes to capture accurate data. The data collected were continuously checked with the participants to confirm that transcriptions were true descriptions and interpretations of every participant’s own perception and experience. Field notes and the digital recording were given to the supervisor to check the allocated themes.
- **Dependability** refers to the findings that will not change if a similar study with the same participants and in a similar context were done at a different time (Polit & Beck 2012:585). The researcher used individual semi-structured, one-to-one individual interview method for all the participants. A digital recorder and field notes were used to ensure an audit trail. Systematic documentation was created by the researcher for the purpose of the audit trail that was available and accessible to the supervisors as proof of all the decisions made throughout the study. The data were analysed and an agreement was reached by the researcher and supervisors with regard to identification of themes.
- **Transferability** was ensured by the researcher by providing thick description of context, the type of sampling method and the characteristics of participants, data collection and analysis.
- **Confirmability** refers to the objectivity or neutrality of the data; there must be an agreement between two or more independent people regarding the data’s relevance and its meaning (Polit & Beck 2012:585). The researcher and the supervisor reduced data to themes after consensus had been reached.

## Results and discussion

The main theme that emerged from the study was: attitude toward natural menopause.

### Attitude toward natural menopause

This theme exposed the attitude of women regarding natural menopause based on their culture, experience and knowledge. Adequate knowledge and better understanding about menopause by women leads to a positive attitude toward this natural stage in life. If there is misunderstanding about reproductive health-related knowledge on menopause, some women may misunderstand the feeling of being in the menopausal state, such as mood swings and irritability, resulting in inadequate acceptance of this normal phenomenon in ageing women and may thus be perceived in a negative light (Adewuyi & Akinade 2010:1778).

#### Positive attitude

Most participants in the study regarded menopause as a natural process of ageing and life changes. The participants understood natural menopause as a cessation of the menstrual circle, resulting in no more reproduction or falling pregnant. The study conducted by Im et al. (2008:548) revealed that most women had a positive attitude toward natural menopause.

African women above 60 years who experienced natural menopause for a longer period, perceived it positively. According to Ramakuela et al. (2014:2), menopause was accepted as a normal phenomenon and menopausal women felt happy about the post-reproductive life stage. Some of the participants said:

‘I think menopause is the time when you become old and you are no longer going to fall pregnant again. It is a natural change of life and ageing. We are living a good life like children. You no longer have a problem of buying some pads. I really enjoy this stage. You can sleep without clothes and not being disturbed.’ (File C/Participant 7, 60 years old)

‘I feel alright with the stage of menopause. I must admit that now is the time I did not start of being in the stage of menopause [*sic*] but I started there, way back, as a lady. Now is the time to be in this stage because everything has its time and I cannot be ever young.’ (File C/Participant 17, 58 years old)

‘I am growing old. The age is catching up with me but I am just happy because I do not have my periods or buy any pads.’ (File D/Participant 1, 63 years old)

As noted from the quotes above, African women with experience, knowledge and who are above 60 years of age accept natural menopause as a normal ageing process. Sociocultural beliefs had no influence on the freedom to discuss natural menopause openly with the researcher, who was not only a woman but also above 50 years of age and within the class of African menopausal women. The African women discussed natural menopause freely without any signs of embarrassment and accepted the physiological changes occurring in their bodies without complaints.

#### Negative attitude

Although most of the participants accepted menopause as a natural process of ageing, some participants who were below the age of 60 were negative, due to about the changes occurring in their bodies after cessation of menstruation. These women highlighted their lack of knowledge about natural menopause because of cultural beliefs, which restricted them from talking about it, as it is regarded as taboo. One participant said:

‘I do not have enough information about menopause. It is a difficult stage because you do not have the experience and knowledge of what to expect. I do not understand why there are changes in my body or life as whole.’ (File D/Participant 11, 52 years old)

The quote above indicates that the participant (52 years old) did not understand the changes occurring in her body and lacked knowledge about the physiological processes associated with female ageing. Below, the quote from another participant:

‘At least, when you were menstruating every month, it prevented you from being ill. But now, my menstruation is no longer coming out and causes a lot of diseases. I am person who is having a lot of blood. Since I stopped to have my monthly period, my blood pressure is high. It can be a problem; you can maybe have … womb cancer. There are lots of diseases which can occur in your body.’ (File C/Participant 15, 49 years old)

Participants below 60 years of age believed that the ‘blood that is not coming out’ (File D/Participant 11, 53 years old) accumulates in their body and cause health problems. The women presented the following signs and symptoms as caused by menopause: headache, backache, painful joints and generalised body pain. Pain was the most common menopausal symptom linked with blood ‘that does not come out’, as shown in the quote below. One of the participants said:

‘I think it is this blood that does not flow, that does not come out. I do not know where this blood goes. I am thinking that maybe it is accumulation of blood in the body that causes pains because the blood does not manufacture babies any longer and no more functioning [*sic*].’ (File D/Participant 11, 53 years old)

Some of the participants in the study related the body changes to changes in their hormones and retained blood resulting in weight gain and skin pigmentation, as revealed below:

‘My skin and body size have changes. The face is dark and wrinkled and not shiny like when I was young. My skin is dry and itching even if I have washed; I think is the hormones and ageing.’ (File D/Participant 4, 55 years old)

‘I gained weight. My face has pimples because of the “blood that does not come out.”’ (File C/Participant 11, 53 years old)

The explanation above indicates that some women lack knowledge about menopause and have contradictory sentiments. This was supported by the study conducted by Mustafa and Sabir (2012:175), who stated that women believe menopause is a natural condition that affects them in a negative manner. The study conducted by Agwu et al. (2008:219) alluded to the fact that some women believed that abstinence from intercourse precipitates menopause and heralds its symptoms.

A widowed participant said:

‘I think menopause is to grow up but my elder sisters, who are older than me, are still menstruating. They are having their husbands. So, I think when you are menstruating, you sleep with a man. I am not sleeping with a man, that it is why menstruation has stopped. The blood stays in your body makes you sick, it must come out every month. It stays under the stomach and at the back and make you feel pains.’ (File D/Participant 4, 46 years old)

This indicates that some of the women below 60 years of age do not understand the causes of menopause because of limited experience and inadequate reproductive health-related knowledge during their menopausal stage and education development regarding menopause and procreation. It is supported by the quote below, which implies that menopause was regarded as taboo to discuss with an elder; there was no sharing of knowledge and experience.

One participant said:

‘I have so many complaints and my mother has six children, but I do not remember her complaining about heat and other diseases. What makes me feel bad is that I did not have my own children.’ (File D/Participant 12, 48 years old)

The study revealed that the attitude of some African menopausal women was negative. Most of the women who expressed a negative attitude toward menopause were below 60 years of age and had limited knowledge related to reproductive health-related knowledge about the natural ageing part of menopause. The women of 60 years old and above understood and accepted menopause as a natural phenomenon in life and part of ageing. This indicates that they accepted menopause more positively than women below the age of 50 years (Robak-Chołubek et al. 2014:666).

The findings of the study provide insight on African women’s perception regarding natural menopause. The more aged the African women, the more knowledgeable and experienced about natural menopause they become. This study revealed that ‘young’ African menopausal women (below the age of 50 years) lack adequate reproductive health-related knowledge about physiological human developmental stages (Robak-Chołubek et al. 2014:666). Inexperience and inadequate health-related knowledge on menopause negatively affects the understanding and perception of natural menopause. African menopausal women need support systems from all relevant stakeholders, including the government, to reduce the cost of curative measures when these women are chronically ill.

## Limitations of the study

The findings of this study were obtained from menopausal women, between the ages 45 and 60 years, in Mamelodi, Tshwane district, therefore they cannot be generalised as these were the perceptions of the women in this particular setting. More studies on women’s cultural perceptions on natural menopause need to be done in South Africa to explore and describe their attitude regarding this natural phenomenon.

## Recommendations

The second objective of the study was to propose recommendations for healthcare providers in all healthcare settings to provide reproductive health-related knowledge regarding human developmental stages for all women in Tshwane district. The participants in this study suggested that they need to be furnished with reproductive health-related knowledge during the visit to healthcare settings. Based on the findings of the study, the researcher has recommendations to clinical practice, nursing education and nursing research with regard to natural menopause.

### Clinical practice

Healthcare providers should provide health education on menopause to women who visit the clinic for consultations and follow-up on chronic medications. The researcher further recommends that Gauteng Province government’s health department, in collaboration with Tshwane district, provide clinic services for menopausal women, which will be managed by their peers to reduce embarrassment and allow for open, free discussion.

### Health education

Tshwane district should include reproductive health and human development, especially female reproductive development, in health education for all female clients in community health clinics. The health education of African women should also include a memorandum of understanding (MoU) with the Tshwane district and the clinics within its jurisdiction.

### Nursing research

The researcher recommends that further studies be conducted on natural menopause for all African menopausal women. The researchers should collaborate with ageing African women in projects which relate to their health issues in order to promote their cooperation and understanding about health-related matters of the ageing African female population.

## Conclusion

The perceptions of women regarding natural menopause revealed that menopause is viewed according to the attitude of the participants, which is influenced by their cultural beliefs and reproductive health knowledge related to menopause, as well as women’s ageing process and development. Some African menopausal women perceived menopause as positive whilst others perceived it as negative, depending on the understanding of the participant respectively. There is a lack of information sharing and limited knowledge about natural menopause because of some cultural sensitivity of reproductive topics and fear of embarrassment. It seems as if the Tshwane district clinics do not provide certain services to women in menopause. These are support systems, such as social networking, and equal opportunities for education in reproductive health and menopause (Thekkedath & Joseph 2009:19). Peer groups for menopausal women in Mamelodi, Tshwane district could be a positive step toward health and wellbeing.

There is a need for community health nurses and other healthcare providers to provide menopausal services such as seminars and health education on menopause to women to reduce the health problems that are related to this phenomenon, by preparing the women in an open, free environment and sharing this natural stage with others. One of the functions of nursing is teaching or educating the public through health education, therefore all nurses should educate the clients at the clinic level as well as during home visits. The South African Nursing Council should ensure that all aspects which are currently overlooked when caring for patients and their families because of changed diet-related life styles which impact on human physiological processes, are somehow revived in the practice of nursing.
